# The AXL receptor tyrosine kinase is associated with adverse prognosis and distant metastasis in esophageal squamous cell carcinoma

**DOI:** 10.18632/oncotarget.9231

**Published:** 2016-05-09

**Authors:** Min-Shu Hsieh, Pei-Wen Yang, Li-Fan Wong, Jang-Ming Lee

**Affiliations:** ^1^ Department of Pathology, National Taiwan University Hospital and National Taiwan University College of Medicine, Taipei, Taiwan; ^2^ Department of Surgery, National Taiwan University Hospital and National Taiwan University College of Medicine, Taipei, Taiwan; ^3^ Graduate Institute of Pathology, College of Medicine, National Taiwan University, Taipei, Taiwan

**Keywords:** esophageal cancer, esophageal squamous cell carcinoma, AXL, HER2, targeted therapy

## Abstract

Esophageal squamous cell carcinoma (ESCC) is a frequently recurrent deadly cancer for which no efficient targeted drug exists. AXL is an adverse prognostic factor in some cancers. Strong clinical evidence to support the prognostic role of AXL in ESCC is lacking. A total of 116 patients diagnosed with operable primary ESCC were enrolled. Both AXL and HER2 expression were detected by immunohistochemistry (IHC) in esophageal tissue and were correlated with the clinical outcome of patients. The efficacy of the AXL targeted drug foretinib was also evaluated in ESCC cells. Expression of AXL was found in about 80 % of ESCC tissue, and was significantly correlated with progression of tumor (P<0.001), increased risk of death (Hazard ratio HR [95 % CI=2.09[1.09-4.04], P=0.028], and distant metastasis (odds ratio OR [95 %CI]=3.96 (1.16-13.60), P=0.029). The adverse clinical impact of AXL was more evident when cumulatively expressed with HER2. In cell model, ESCC cells were more sensitive to AXL inhibitor foretinib than to the HER2 inhibitor lapatinib. Meanwhile, the AXL inhibitor foretinib showed a synergistic effect with HER2 inhibitors and the potential to overcome drug resistance to lapatinib. We thus concluded that AXL is a strong adverse prognostic factor for ESCC. Therapeutic agents targeting AXL have great potential to improve prognosis of ESCC patients.

## INTRODUCTION

Esophageal cancer is a fast-growing and deadly disease. It presents most often as esophageal squamous cell carcinoma (ESCC), which accounts for 95 % of esophageal cancer worldwide [1–2]. The standard treatment for locally advanced esophageal cancer is concurrent chemoradiotherapy (CCRT) with or without surgery. Patients with esophageal cancer enjoy better survival once they have had a good response to neoadjuvant therapy [3]. Unfortunately, there is no effective targeted therapeutic strategy available for ESCC and the prognosis for esophageal cancer is relatively poor with an average 5-year survival rate of less than 20 % [1, 4–5].

HER2/Neu belongs to the epidermal growth factor receptor (EGFR/ErbB) family. It is an important drug target for cancers, including breast cancer and gastroesophageal (GE) junction adenocarcinoma [6–7]. HER2 inhibition in combination with cisplatin-based chemotherapy has been demonstrated to significantly improve response rate and overall or progression-free survival in patients with adenocarcinoma of the gastro-esophageal junction or the stomach who over-express HER2 [7]. In studies of ESCC, however, the rate of HER2 over-expression has been reported to be less than 10 % [8–10] and the efficacy of HER2 targeted drugs in ESCC has not been demonstrated.

AXL (also call Ark or Ufo) is a receptor tyrosine kinase belonging to the Tyro3/Axl/Mer (TAM) family [11]. Activation of AXL receptors initiates signaling pathways involved in multiple cellular events, including cell survival, anti-apoptosis, proliferation, migration, and cytokine production [12]. AXL is ubiquitously expressed in cells and organs, and its over-expression has been reported in a wide array of human cancers, including breast [13], lung [14], liver [15], colon [16], and esophageal adenocarcinoma [17]. It has also been found to be an important biomarker for prognosis of cancer. Up-regulation of AXL is associated with poor survival of breast cancer [18], lung adenocarcinoma [14], and acute myeloid leukemia [19].

AXL has been reported as an adverse prognostic factor and a therapeutic target in esophageal adenocarcinoma (EAC) [20]. Knockdown of AXL inhibited invasion and migration of EAC cells, and the AXL inhibitor R428 significantly reduced invasion and migration of EAC cells [20]. AXL also mediates TRAIL (tumor necrosis factor-related apoptosis-inducing ligand) and cisplatin resistance in EAC [21–23]. The crucial role of AXL in tumorigenesis and drug resistance in ESCC has been clearly demonstrated only very recently [24–25]. AXL was found to be consistently over-expressed in ESCC cells and human tumor samples [24]. Knockdown of AXL expression was shown to inhibit cell proliferation, survival, migration and invasion both *in vitro* and *in vivo* [24]. The tumorigenic function of AXL is mediated by activation of the Akt/NF-κB and Akt/GSK3β pathways [24]. Over-expression of AXL also mediates resistance to treatment with the phosphoinositide -3-kinase-alpha (PI3Kα) inhibitor BYL719 by activating the EGFR/PKC/mTOR axis in ESCC [25]. Resistance to PI3Kα can be reversed by combined treatment with AXL, EGFR, and PKC inhibitors [25].

HER2-targeted agents, including trastuzumab and lapatinib, are a promising targeted therapy, especially in treating breast cancer. Over-expression of AXL has been shown to be a novel mechanism of acquired resistance to HER2-targeted agents in lapatinib-resistant, HER2-positive breast cancer clones [26]. Foretinib (XL880, GSK1363089), an oral multi-kinase inhibitor acting on AXL, c-Met, RON and VEGFR-2, can restore sensitivities to lapatinib and trastuzumab in resistant cells [26]. Synergistic effects of foretinib with HER-targets have been demonstrated in MET and HER1/2 co-activated cells [27]. Meanwhile, the AXL inhibitor BMS777607 and HER2 inhibitor lapatinib exhibit a synergistic cytotoxic effect in breast and ovarian cancer cells [28]. However, the prognostic role of co-expression of AXL and HER2 in cancer cells has hardly been investigated.

Although the molecular function of AXL in ESCC has been demonstrated, clinically there is still a lack of evidence to support the prognostic significance of AXL in ESCC. In our study, we investigated the prognostic relevance of AXL and HER2 expression in operable ESCC patients (116 cases) and the efficacy of the AXL inhibitor, foretinib [29], in wild type and HER2-resistant ESCC cells.

## RESULTS

A total of 116 patients who were diagnosed with ESCC and received surgical resection were enrolled in this study. In this cohort, 107 patients (92.2 %) were male and 1 (0.9 %), 25 (21.5%), 54 (46.6%), and 36 (31.0%) were diagnosed with pathologic stage 0, I, II, and III disease, respectively. A total of 75 patients (64.6 %) were treated with CCRT (concurrent chemoradiotherapy) (Table 1). As expected, both pathologic stage and T-stage (tumor stage) were significantly correlated with both survival and recurrence status of patients (P=0.001 for pathologic stage and survival; P<0.001 for pathologic stage and recurrence; P=0.003 for T-stage and survival and P=0.004 for T-stage and recurrence, Table 1). There were also statistically significant differences in the distributions of sex and CCRT treatment by survival and recurrence status (P=0.004 and P=0.023 respectively for survival; P=0.001 and P=0.013 respectively for recurrence, Table 1). A total of 93 patients (80.2 %) exhibited positive expression of AXL in tumor tissue. Significant differences in mortality and disease recurrence status were also observed between AXL-positive patients and AXL-negative patients (Table 1).

**Table 1:** Demographic and clinical characteristics of ESCC patients by survival and recurrence status

		Survival			Recurrence		
Variables	Total	Alive	Dead	*p*-value	no recurrence	recurrence	*p*-value
		26 (22.4)	90 (77.6)		21 (18.1)	95 (81.9)	
**Age (years)**				0.299			0.104
<40	9(7.8)	4 (44.4)	5 (55.6)		4 (44.4)	5 (55.6)	
40-60	61 (52.2)	13 (21.3)	48 (78.7)		9 (14.8)	52 (85.2)	
>60	46 (39.7)	9 (19.6)	37 (80.4)		8 (17.4)	38 (82.6)	
**Sex**				**0.004**			**0.001**
Male	107 (92.2)	20 (18.7)	87 (81.3)		15 (14.0)	92 (86.0)	
Female	9 (7.8)	6 (66.7)	3 (33.3)		6 (66.7)	3 (33.3)	
**Stage**				**0.001**			**<0.001**
0	1 (0.9)	1 (100)	0 (0)		1 (100)	0 (0)	
I	25 (21.5)	12 (48.0)	13 (52.0)		11 (44.0)	14 (56.0)	
II	54 (46.6)	10 (18.5)	44 (81.5)		7 (13.0)	47 (87.0)	
III	36 (31.0)	3 (8.3)	33 (91.7)		2 (5.6)	34 (94.4)	
**T-stage**				**0.003**			**0.004**
0	1 (0.9)	1 (100)	0 (0)		1 (100)	0 (0)	
1	32 (27.6)	14 (43.8)	18 (56.3)		12 (37.5)	20 (62.5)	
2	33 (28.4)	5 (15.2)	28 (84.8)		4 (12.1)	29 (87.9)	
3	44 (37.9)	6 (13.6)	38 (86.4)		4 (9.1)	40 (90.9)	
4	6 (5.2)	0 (0)	6 (100.0)		0 (0)	6 (100.0)	
**N-stage**				0.195			0.069
0	65 (56.0)	19 (29.2)	46 (70.8)		17 (26.2)	48 (73.8)	
1	45 (38.8)	6 (13.3)	39 (86.7)		4 (8.9)	41 (91.1)	
2	5 (4.3)	1 (20.0)	4 (80.0)		0 (0)	5 (100)	
3	1 (0.9)	0 (0)	1 (100)		0 (0)	1 (100)	
**Tumor location**				0.409			0.424
Upper	24 (20.7)	3 (12.5)	21 (87.5)		2 (8.3)	22 (91.7)	
Middle	48 (41.4)	11 (22.9)	37 (77.1)		10 (20.8)	38 (79.2)	
Lower	44 (37.9)	12 (27.3)	32 (72.7)		9 (20.5)	35 (79.5)	
**CCRT**				**0.023**			**0.013**
No	38 (32.8)	14 (36.8)	24 (63.2)		12 (31.6)	26 (68.4)	
Yes	75 (64.6)	11 (14.7)	64 (85.3)		8 (10.7)	67 (89.3)	
CT or RT	3 (2.6)	1 (33.3)	2 (66.7)		1 (33.3)	2 (66.7)	
**AXL expression**				**0.002**			**0.020**
negative	23 (19.8)	11 (47.8)	12 (52.2)		8 (34.8)	15 (65.2)	
positive	93 (80.2)	15 (16.1)	78 (83.9)		13 (14.0)	80 (86.0)	

**Figure 1:**
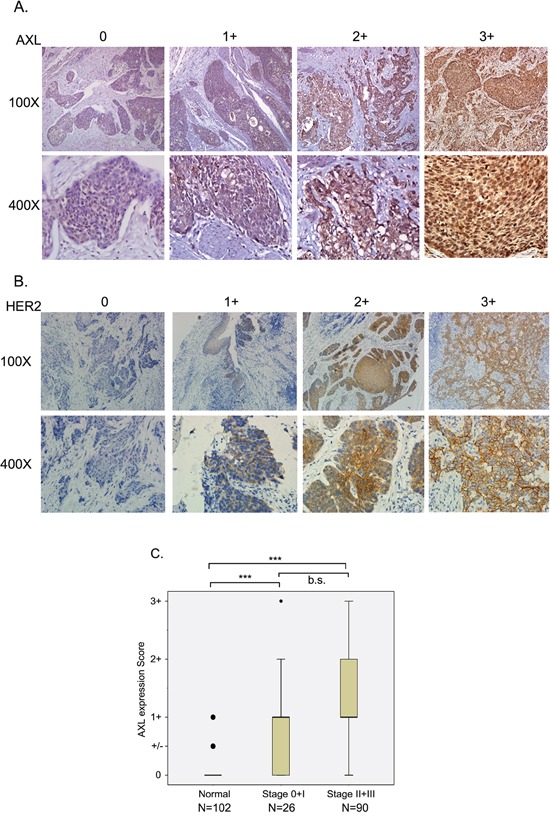
Expression of AXL and HER2 in ESCC tissue specimens Immuno-histochemical analysis of AXL **A.** or HER2 **B.** expression in ESCC tissues is shown by expression level. **A.** Score 0, no reactivity in any tumor cell; Score 1+, tumor cells cluster with a faint reactivity irrespective of tumor cells stained; Score 2+, tumor cells cluster with a moderate reactivity irrespective of tumor cells stained; Score 3+, tumor cells cluster with a diffuse and strong reactivity. **B.** Score 0, no reactivity or no membranous reactivity in any tumor cell; Score 1+, tumor cells with a faint or barely perceptible membranous reactivity irrespective of percentage of tumor cells stained; Score 2+, tumor cells cluster with a weak-to-moderate complete membranous reactivity irrespective of percentage of tumor cells stained; Score 3+, tumor cells cluster with a strong complete membranous reactivity irrespective percentage of tumor cells stained. **C.** AXL expression level was analyzed in adjacent non-cancerous (normal), stage 0 and 1, and stage II and III ESCC tissues. ***, P<0.001; b.s., borderline significance.

To analyze the correlation between AXL expression and the prognosis of ESCC patients, we detected AXL expression in cancerous and non-cancerous esophageal tissues by IHC and correlated it with overall survival (OS) and progression-free survival (PFS) by multivariate Cox regression analysis. Expression levels were scored as 0, 1+, 2+, and 3+ (Figure 1A). AXL expression was absent in 19.8 % of the ESCC tissue samples (score=0, 23/116); 48.3 % (56/116) of ESCC tissues showed faint reactivity (score=1+); 24.1 % (28/116) moderate reactivity (score=2+); and 7.8 % (9/116) diffuse and strong reactivity (score=3) (Figure 1A and Table 2). Notably, the expression levels of AXL were significantly elevated in cancerous tissue compared to non-cancerous tissue (P<0.001, one-way ANOVA, Scheffe's test, Figure 1C). Advanced tumor tissue (stage II and III) also showed increased expression of AXL compared to levels in early-stage tumor tissues, but the increase did not reach statistical significance (P=0.102, one-way ANOVA, Scheffe's test, Figure 1C). Expression of AXL was significantly associated with increased risk of death (HR [95 % CI]=2.09 [1.09-4.04], P=0.028, Table 2) and borderline significantly correlated with disease progression (P=0.062, Table 2). Patients with strong AXL expression (score=3+) exhibited about a 3-fold higher risk of mortality and disease progression (HR [95 % CI]=2.98[1.17-7.58], P=0.022 for OS; HR [95 % CI]=2.79 [1.14-6.84], P=0.025 for PFS, Table 2). Faint expression of AXL in non-cancerous (normal, 1+, 5.6 % [5/89]) esophageal tissue also significantly correlated with increased risks of death and disease recurrence (HR [95 % CI]=3.63 [1.29-10.29], P=0.015 for OS; HR [95 % CI]=2.72 [1.01-7.33], P=0.048 for PFS, Table 2).

**Table 2:** Association of AXL and HER2 expression with overall and progression-free survival of ESCC patients under multivariate analysis

Variables	N	Overall survival HR (95 % CI)	*P-value	Progression-free survival HR (95 % CI)	*P-value
**Tumor_AXL expression**					
0	23 (19.8)	1		1	
1+	56 (48.3)	2.24 (1.14 (4.42)	**0.020**	1.82 (0.99-3.35)	0.056
2+	28 (24.1)	1.60 (0.75-3.39)	0.224	1.42 (0.72-2.81)	0.318
3+	9 (7.8)	2.98 (1.17-7.58)	**0.022**	2.79 (1.14-6.84)	**0.025**
Negative	23 (19.8)	1		1	
Positive	93 (80.2)	2.09 (1.09-4.04)	**0.028**	1.75 (0.97-3.16)	0.062
**Normal_AXL expression**					
0	73 (82.0)	1		1	
+/−	11 (12.4)	1.34 (0.64-2.80)	0.444	1.45 (0.71-2.95)	0.306
1+	5 (5.6)	3.63 (1.29-10.29)	**0.015**	2.72 (1.01-7.33)	**0.048**
**Tumor_HER2 expression**					
0	90 (81.8)	1		1	
1	17 (15.5)	1.47 (0.79-2.76)	0.228	1.54 (0.83-2.86)	0.173
2	2 (1.8)	3.00 (0.69-13.12)	0.144	3.19 (0.73-13.90)	0.123
3	1 (0.9)	2.20 (0.27-17.73)	0.458	1.42 (0.18-11.23)	0.740
Negative	90 (81.8)	1		1	
Positive	20 (18.2)	1.59 (0.89-2.86)	0.118	1.62 (0.91-2.89)	0.101
**Tumor_AXL_HER2_expression**					
AXL (−) and HER2 (−)	17 (15.5)	1		1	
AXL (+) or HER2 (+)	77 (70.0)	1.87 (0.91-3.88)	**0.091**	1.54 (0.79-3.00)	0.203
AXL (+) and HER2 (+)	16 (14.5)	3.43 (1.40-8.42)	**0.007**	3.19 (1.37-7.45)	**0.007**

*adjusted for stage, age, sex and CCRT

The functional interplay between AXL and HER2 (ErbB2/Neu) has been suggested in previous studies [20, 26]. We also analyzed the prognostic relevance of HER2 in our ESCC subjects (N=110) by IHC. Expression levels of HER2 in ESCC were scored as 0, 1, 2, and 3 (Figure 1B). Only 18.2 % (20/110) of ESCC tissues had positive IHC staining and expression of HER2 did not correlate with either risk of death or of recurrence (Table 2). Cumulative analysis of the effects of expression of AXL and HER2 on prognosis revealed that co-expression of AXL and HER2 notably increased the hazards of both death and recurrence by more than 3-fold (HR [95 % CI]=3.43 [1.40-8.42], P=0.007 for OS; HR [95 % CI]=3.19[1.37-7.45], P=0.007 for PFS, Table 2).

**Figure 2:**
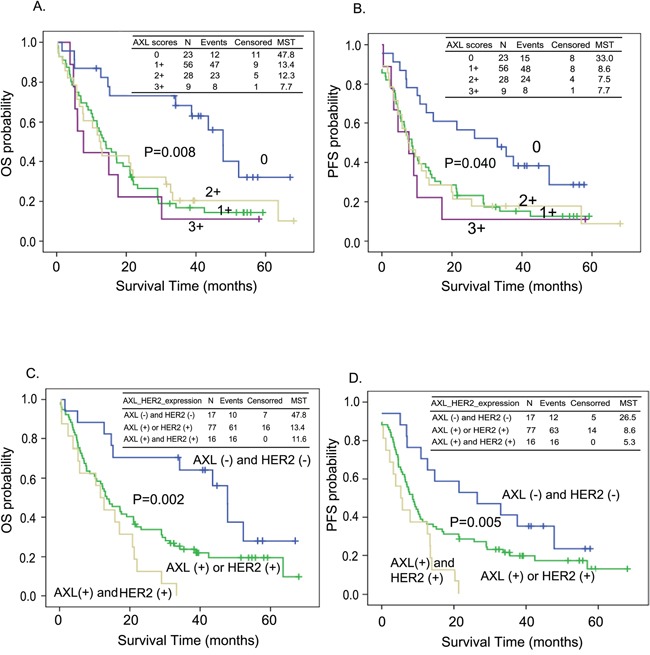
Kaplan-Meier estimates of overall survival (OS, A and C) or progression-free survival (PFS, B and D) by the expression level of AXL only **A.** and **B.** or both AXL and HER2 **C.** and **D.** MST: median survival time (months).

The prognostic relevance of AXL and HER2 in ESCC were also analyzed with Kaplan-Meier estimates. Both OS and PFS differed significantly between patients with different levels of AXL expression (median survival time [MST] = 47.8 vs. 13.4 vs. 12.3 vs. 7.7 months in tissues expressing levels 0, 1+, 2+, 3+ of AXL, log-rank P=0.008 for OS, Figure 2A; MST= 33.0 vs. 8.6 vs. 7.5 vs. 7.7 months in tissues expressing levels 0, 1+, 2+, 3+ of AXL, log-rank P=0.040 for PFS, Figure 2B). Patients with both AXL and HER2 expression exhibited significantly shorter OS and PFS (MST= 47.8 vs. 13.4 vs. 11.6 months in the AXL (−) and HER2 (−), AXL (+) or HER2 (+), and HER2 (+) and AXL (+) subgroups, log-rank P=0.002 for OS, Figure 2C; MST= 26.5 vs. 8.6 vs. 5.3 months in the AXL (−) and HER2 (−), AXL (+) or HER2 (+), and HER2 (+) and AXL (+) subgroups, log-rank P=0.005 for PFS, Figure 2D).

Patients with expression of AXL in their tumor tissue showed significantly increased risk of distant metastasis, a 3.96 fold increase, compared to patients without AXL in their tumor tissue (OR [95 %CI]=3.96 (1.16-13.60), P=0.029, Table 3). Co-expression of AXL and HER2 also increased the risk of recurrence compared to patients with neither AXL nor HER2 expression, however, without reaching statistical significance (OR [95 % CI]=4.11(0.59-28.46), P=0.152, Table 3). Interestingly, the recurrence pattern of patients differed significantly according to expression of AXL in tumor tissue. Patients testing positive for AXL staining exhibited a significantly increased rate of distant metastasis (the proportion with no recurrence, local recurrence, and distant metastasis were 44.4 % vs. 16.7 % vs. 38.9 % in AXL-negative patients and 18.1 % vs. 8.3 % vs. 73.6 % in AXL-positive patients, respectively, P=0.017, Table 4). HER2 expression, however, was not associated with a different recurrence pattern (P=0.247, Table 4). Co-expression of AXL and HER2 were also associated with an increased incidence of distant metastasis compared to patients with neither AXL nor HER expression (the proportion with no recurrence, local recurrence, and distant metastasis were 1.7 % vs. 16.7 % vs. 41.7% in the AXL (−) and HER2 (−) group and 0 % vs. 25 % vs. 75 % in the AXL (+) and HER2 (+) group, respectively, P=0.012, Table 4). That is, both AXL expression alone and also AXL co-expression with HER2 were associated with increased risk of death and tumor recurrence in patients with ESCC.

**Table 3:** Association of AXL and HER2 expression with risk of distant metastasis of ESCC patients under multivariate analysis

Variables	N	Distant metastasis	*P-value
**Tumor_AXL expression**			
Negative	18	1	
Positive	72	3.96 (1.16-13.60)	**0.029**
**Tumor_AXL_HER2_expression**			
AXL (−) and HER2 (−)	12	1	
AXL (+) or HER2 (+)	60	3.27 (0.80-13.34)	0.099
AXL (+) and HER2 (+)	12	4.11 (0.59-28.46)	0.152

*adjusted for stage and CCRT

**Figure 3:**
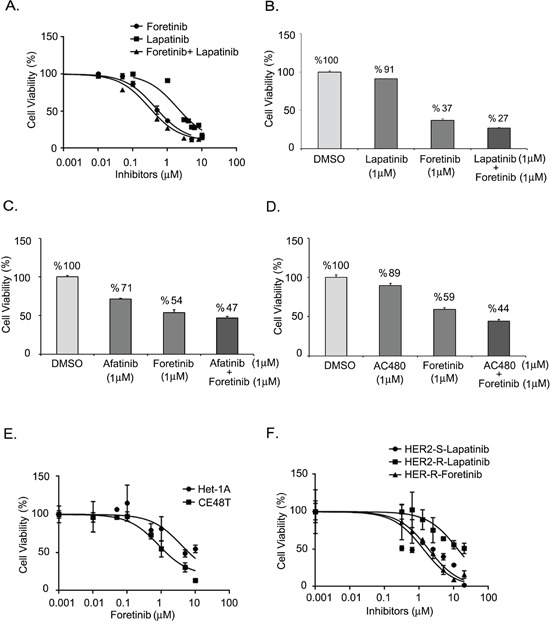
Effect of AXL and HER2 inhibitors on ESCC cells **A.** Dose-inhibition curves of CE48T ESCC cells in response to indicated concentration of lapatinib, foretinib, or foretinib plus lapatinib. The respective IC_50_ values were 1.891 μM, 0.443 μM and 0.296 μM. **B-D.** Synergistic effects of foretinib and HER2 inhibitors on the cytotoxicities of ESCC. Cell viability of ESCC cells treated by 1 μM of foretinib, or 1 μM of HER2 inhibitors, or foretinib plus HER2 inhibitor. The HER2 inhibitors included 1 μM of lapatinib **B.** or 1 μM of afatinib **C.** or 1 μM of AC480 **D. E.** Relative cell viabilities of Het-1A and CE48T cells treated with indicated concentration of foretinib. The IC_50_ values were 3.836 μM vs. 0.823 μM for Het-1A and CE48T cells respectively. **F.** Dose-response curves for HER2-sensitive (HER2-S) and HER2-resistant (HER2-R) ESCC cells in response to increased concentrations of lapatinib or foretinib.

In the cell model, we used human ESCC cells cultured from the CE48T cell line to analyze the efficacy of lapatinib, a HER2 inhibitor, and of foretinib (GSK1363089), a multikinase inhibitor of AXL, c-Met and VEGFR-2 [29]. Figure 3A shows the relative cell viabilities of the ESCC cells in response to the indicated concentrations of inhibitors. ESCC cells were more sensitive to foretinib or foretinb plus lapatinib than to lapatinib alone. The IC_50_ values were 1.891 μM, 0.443 μM and 0.296 μM of lapatinib, foretinib, and lapatinib plus foretinib, respectively (Figure 3A). Synergistic effects of lapatinib and foretinib were demonstrated. The cell viabilities were decreased to 91% and 37% in cells treated with 1 μM of lapatinib and 1 μM of foretinib respectively. The viability was further inhibited to 27 % by combining 1 μM of lapatinib and 1 μM foretinib (Figure 3B). The efficacies of combined targeted therapies in ESCC cells were also examined in two other HER2-targeted drugs, afatinib [38] and AC480 (BMS599626) [39] (Figure 3C and 3D). Cytotoxicity of foretinib was also increased by treatment with either afatinib (Figure 3C) or AC480 (Figure 3D).

**Figure 4:**
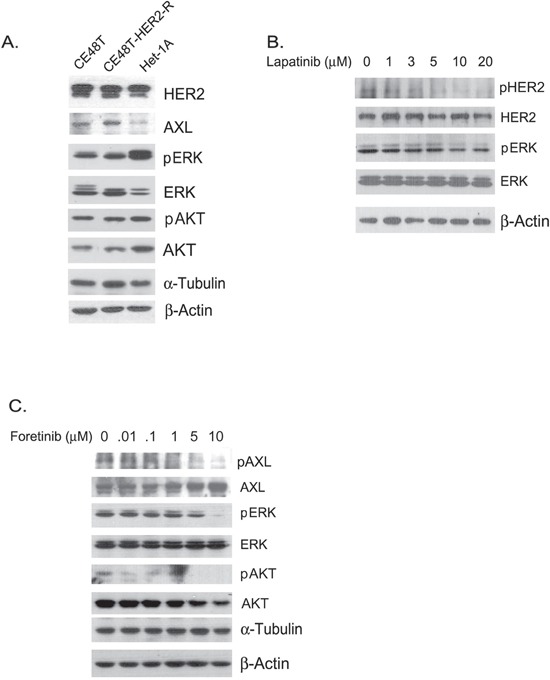
**A.** Protein expression profiles of CE48T, HER2-resistant CE48T cells (CE48T-HER2-R) and Het-1A cells. α-Tubulin served as a loading control for phospho-ERK (pERK) and ERK whereas β-Actin was used as a loading control for AXL, HER2, phospho-AKT (pAKT), and AKT. **B.** Phospho-HER2 (pHER2), HER2, pERK, and ERK expression in ESCC cells treated with indicated amounts of lapatinib for 24 hours. β-Actin served as a loading control. **C.** Phospho-AXL (pAXL), AXL, pERK, ERK, pAKT, and AKT expression in ESCC cells treated with the indicated amounts of foretinib for 24 hours. α-Tubulin served as a loading control for pERK and ERK; β-Actin served as a loading control for pAXL, AXL, pAKT, and AKT.

We also evaluated the sensitivity to foretinib in a non-cancerous esophageal cell line, Het-1A (Figure 3E). The IC_50_ of foretinib was 3.836 μM in Het-1A cells, which was more than 4-fold higher than the value in ESCC cells (0.823 μM). Lapatinib-resistant (HER2 resistant) sub-cells were selected from CE48T cells (Figure 3F) and the IC_50_ value of lapatinib for these cells was measured to be 10 times greater than for parental cells (IC_50=_ 12.980 μM vs. 1.321 μM in HER2 resistant cells and in parental HER2 sensitive cells respectively). Interestingly, the HER2 resistant ESCC cells displayed sensitivity to foretinib (IC_50=_ 1.856 μM). Notably, AXL expression was hardly detected in Het-1A normal esophageal cells and was increased in HER2-resistant cells compared to the expression level in parental ESCC cells (Figure 4A). The activation profiles of ERK (extracellular signal-regulated protein kinase) and AKT were also analyzed. The levels of phospho-ERK(pERK) and phospho-AKT (pAKT) were similar in parental and HER2-resistant CE48T cells (Figure 4A).

The activation profiles of downstream factors in response to lapatinib and foretinib in ESCC were analyzed. As expected, lapatinib dose-dependently and time-dependently inhibited tyrosine phosphorylation of ERK in ESCC cells (Figure 4B and Figure S1). Phosphorylation of HER2 also decreased with increased doses of lapatinib, which revealed the specific inhibition of HER2 activation in ESCC cells (Figure 4B). Oncogenic function of AXL has been demonstrated to function through activating Akt/NF-κB and Akt/GSK3β pathways [24]. In agreement with a previous report, we demonstrated that higher doses of foretinib inhibited phosphorylation of Akt in ESCC cells (Figure 4C). However, total AKT also decreased with increased amounts of foretinib (Figure 4C). Meanwhile, phosphorylation levels of AXL and ERK were also reduced with elevated amounts of foretinib (Figure 4C). Thus, foretinib might induce cytotoxicity of ESCC cells through specific inhibition of AXL activation and of the signaling cascades of PI3K/AKT and Ras/ERK.

## DISCUSSION

ESCC is a deadly cancer with poor prognosis and no clinically approved targeted therapy. The EGFR (epidermal growth factor receptor)-targeted drugs, gefitinib (Iressa) and erlotinib (Tarceva) were evaluated in esophageal cancer in phase II and phase III studies [42–45]. Other targeting agents have been studied for treatment of ESCC, though most have been restricted to pre-clinical tests or phase I studies [46]. A growing body of studies has demonstrated the promise of AXL as a novel target for cancer targeted therapy, including in esophageal cancer. In our current study, we provide further clinical evidence of the oncogenic role of AXL in ESCC. We found that AXL expression was significantly elevated in tumor tissues and associated with pathological stage (Figure 1). Over-expression of AXL increased risk of death (Table 2 and Figure 2), and distant metastasis of ESCC (Table 3 and 4). Patients positive for AXL have about a 2-fold greater hazard of death and about a 4-fold increased risk of disease recurrence. The median survival time was also drastically decreased from 47 months to less than 14 months.

**Table 4:** Recurrence patterns of patients with different AXL and HER2 expression profiles

Variables	N	Recurrence pattern			*p*-value
		No recurrence	Local recurrence	Distant metastasis	
		21 (23.3)	9 (10.0)	60 (66.7)	
**Tumor_AXL**					**0.017**
Negative	18 (20)	8 (44.4)	3 (16.7)	7 (38.9)	
Positive	72 (80)	13 (18.1)	6 (8.3)	53 (73.6)	
**Tumor_HER2**					0.247
Negative	68 (81.0)	17 (25.0)	5 (7.4)	46 (67.6)	
Positive	18 (19.0)	2 (12.5)	3 (18.8)	11 (68.8)	
**Tumor_AXL and HER2**					**0.012**
AXL (−) and HER2 (−)	12 (14.3)	5 (41.7)	2 (16.7)	5 (41.7)	
AXL (+) or HER2 (+)	60 (71.4)	14 (23.3)	3 (5.0)	43 (71.7)	
AXL (+) and HER2 (+)	12 (14.3)	0 (0)	3 (25.0)	9 (75.0)	

In our operable ESCC patients, the rate of positive AXL expression in ESCC tissue was about 80 %, which was markedly higher than in adjacent normal esophageal tissue (5.6 %). AXL expression has been found to be induced by chemotherapy drugs in U937 acute myeloid leukemia cells [47]. About 65 % of our patients were treated with operative neoadjuvant CCRT, which makes reasonable the speculation that AXL expression may have been induced by CCRT. However, the rate of AXL-positive samples were not significantly different between patients who received CCRT and those who did not (positive and negative rates were 84.2 % vs. 15.8 % and 78.2 % vs. 21.8 % in the CCRT [−] and CCRT [+] groups respectively, P=0.446, Table S1). CCRT treatment did not correlate with the expression of HER2 in ESCC either (P=1.000, Table S1). Thus, CCRT was not a major factor in inducing the expression of AXL or HER2 in ESCC tissues.

AXL expression has been found to be induced by epithelial-mesenchymal transition (EMT) and to correlate with induction of EMT in breast cancer [18, 48] and in ESCC [49]. EMT is a critical factor in promoting metastasis. Our results demonstrated that AXL expression significantly correlated with incidence of distant metastasis, providing strong clinical support for the crucial role of AXL in ESCC metastasis.

AXL has also been suggested as a downstream effector of transforming growth factor-beta 1 (TGF-β1) during langerhans cell differentiation [50]. In hepatocellular carcinoma (HCC), the molecular collaboration of AXL/14-3-3ζ and TGF-β/Smad signaling on cancer progression has been demonstrated [51]. TGF-β1 is an upstream factor of EMT [52] and has been shown to be induced by environmental carcinogens, such as tobacco smoking [53], alcohol drinking [54] and betel nut chewing [55–56]. Incidence of ESCC is well-known to highly correlate with these environmental carcinogens. Among our study population, over 90 % of patients have at least one of these unfavorable habits. Interestingly, in our patients, 90.5 % of betel nut chewers were positive for AXL in tumor tissue, which was significantly higher than in non-chewers (P=0.029, data not shown). Such results suggest that these environmental carcinogens might increase the level of TGF-β1 and thus contribute to the over-expression of AXL in esophageal tissue.

Expression of HER2 is highly correlated with the prognosis of esophageal adenocarcinom [6], while the prognostic role in ESCC has hardly been investigated. We found that about 20 % of our patients were positive for HER2. Even though expression of HER2 alone did not associate with the clinical outcome of ESCC, cumulative expression of AXL and HER2 showed significant clinical impact on ESCC. AXL expression was increased in HER2-resistant ESCC cells compared to expression in HER2-sensitive ESCC cells revealing the interplay of AXL and HER2 in ESCC.

The activity of foretinib was demonstrated in a phase II trial in patients with advanced papillary renal cell carcinoma (PRCC), especially in those with germline *MET* mutations [57]. Because c-Met is also an adverse prognostic factor for ESCC [58], we suggest foretinib has great potential for ESCC targeted therapy in patients over-expressing AXL or c-Met. The synergistic cytotoxicity of foretinib with HER2 inhibitors, including lapatinib, afatinib, and AC480 have also been demonstrated in ESCC cells. Combination therapy of AXL and HER2 inhibitors is, therefore, a possible direction in ESCC patients co-overexpressing AXL and HER2.

Collectively, our results provide clinical evidence that AXL is a strong adverse prognostic factor, which is significantly correlated with pathological stage, overall survival, and distant metastasis of operable ESCC. Therapeutic agents targeting AXL, therefore, have great potential to improve the clinical outcome of operable ESCC.

## MATERIALS AND METHODS

### Study population

Our study subjects were collected in the pathological and surgical department of National Taiwan University Hospital from 2005 to 2013. The consent procedure of the clinical study was approved by the Research Ethics Committee of National Taiwan University Hospital (201402056RINA). The inclusion criteria were patients aged above 20 years who were histologically confirmed with primary ESCC and received surgical resection. The exclusion criteria were pregnant women, those unwilling to give informed consent, and those without complete clinical records. Surgical resection of esophagectomy with two or three field lymph node dissection and esophageal reconstruction were performed in all of the recruited patients.

CCRT was administered to patients with advanced TNM stages (IIb or more advanced) diagnosed by endoscopic ultrasound or computed tomography before surgery. The CCRT procedures were described in a previous study [30–31]. Information regarding the demographics, disease characteristics (tumor location, TNM stage), histology, clinical treatments, recurrence status, and survival were obtained from the Tumor Registry of the National Taiwan University Hospital and/or medical chart-review. The TNM staging of the patients receiving CCRT was re-staged according to the pathological reports after surgery [32]. Overall survival duration (OS) was defined as the interval between esophagectomy and either death from disease or last follow-up. Progression-free survival (PFS) was defined as the interval between esophagectomy (complete resection) and detection of local recurrence or distant metastasis of the tumor.

### Immunohistochemistry (IHC)

Formalin-fixed paraffin-embedded (FFPE) blocks with ESCC and adjacent non-tumor tissue samples were obtained from the Department of Pathology in our hospital. Briefly, FFPE tissue sections (4 μm) were dewaxed and rehydrated. After antigen retrieval at 37°C for 2 hours in a Ventana BenchMark XT processor (Ventana, Tucson, AZ), tissue sections on the slides were incubated with primary antibodies. After reincubation with polymer-horseradish peroxidase reagent, the sections were visualized with Ventana ultraview DAB (diaminobenzidine 3,3′ tetrahydrochloride) detection kit according to the manufacturer's protocol. The primary antibodies used were a rabbit polyclonal antibody against AXL (1:100, ab72069, abcam) and HER2 (ready- to-use, DAKO). The tissue slides were then counterstained with hematoxylin.

### Cell culture

CE48T/VGH (CE48T, BCRC 60165), a human ESCC cell line derived from a 57 year-old Taiwanese man [33–34], was cultured in RPMI complete medium. Het-1A, a non-tumorigenic primary esophageal squamous cell line, was transformed by SV40 T antigen [35] and was cultured on CellBind dishes (Corning) in BEGM medium (Lonza). All the cells were cultured at 37°C in an incubator containing 5% CO2.

### Isolation of HER2-resistant ESCC cells

HER2-resistant ESCC sub-cells were selected by repeated treatment with the HER2 inhibitor lapatinib (Tykerb, synthesized by Selleckchem, S2111) [36] of different concentrations (5 to 20 μM/L). Briefly, the 50 % and 90 % inhibition concentrations (IC_50_ and IC_90_) of lapatinib for a 72 hour treatment period were determined. The parental cells were then treated with the IC_90_ dose for 24 hours. After removing the medium, the cells were then maintained and regrown in medium containing the IC50 dose of lapatinib. When cells covered 60-70 % of the dish surface, the cells were re-incubated with an increased concentration (1.5 fold) of the drug. Cells resistant to *20* μM/L of lapatinib were harvested and were subjected to cell viability assay.

### Cell viability assay

The CE48T/VGH cells, lapatinib-resistant sub-cells and Het-1A cells were plated on 96-well plates (8000 cells/well) and treated with the indicated amounts of AXL inhibitor, foretinib [37] (GSK1363089, provided by Santa Cruz Biotechnology, Inc. SC-364492) or HER2 inhibitors. The HER2 inhibitors included lapatinib, afatinib [38] (Tovok, provided by Boehringer Ingelheim, Taiwan), and AC480 [39] (BMS599626, synthesized by Selleckchem, S1056). After incubation for 72 hours, cell survival was determined by MTT assay as described previously [40–41].

### Protein extraction and western blotting

To detect protein expression in the esophageal cells, total protein was extracted from the cells by RIPA buffer (150 mM NaCl, 50 mM Tris–HCl [pH 7.5], 1 % Igepal-CA630, 0.5 % sodium deoxycholate, 0.1 % SDS, 50 mM NaF, 1 mM Na3VaO4, and complete protease inhibitor cocktail). Protein expression was analyzed by SDS-PAGE and western blotting with specific antibodies as described previously [40–41]. The primary antibodies for protein detection were anti-AXL rabbit polyclonal antibody (pAb) (ab72069, Abcam), anti-phospho-AXL (Tyr702, D12B2) rabbit monoclonal antibody (mAb) (#5724, Cell Signaling Technology), anti-HER2 rabbit antibody (#2242, Cell signaling), anti-phospho -HER2/ErbB2 (Tyr1221/1222, 6B12) pAb (#2243, Cell Signaling Technology), anti-Actin mAb (clone C4, Millipore), anti-ERK 1 (K-23) (Santa Cruz), anti-phospho-p44/42 Erk1/2 (Thr202/Tyr204) mAb (#4370, Cell Signaling Technology), anti-Akt antibody (#9272, Cell Signaling Technology), anti-phospho-Akt (Ser473) (#9271, Cell Signaling Technology), and anti-α-Tubulin (DM1A, Abcam).

### Statistical analysis

The distribution of demographic variables, clinical characteristics, and indicated protein expression levels among the subgroups with different prognostic results were compared using a Pearson χ^2^ test or Fisher exact test (N≤ 5). Protein expression levels of esophageal tissues among non-tumor and tumor tissue of different stages were analyzed by one-way ANOVA and box-plot. Multivariate Cox regression analysis was used to evaluate the hazard ratios (HRs) of death and disease progression adjusted for potential significant clinical covariates and indicated protein expression. The odds ratios (ORs) obtained from logistic regression were used to describe association of distant metastasis and protein expression profiles. Data were expressed as mean value and 95% confidence interval (CI). Crude correlations between genotypes and OS or PFS were assessed using the Kaplan-Meier survival function and compared using the log-rank test. All statistical analyses were conducted with SPSS 17.0 (SPSS Institute, Chicago, IL). A *p*-value ≤ 0.05 was considered statistically significant. Sigmoidal dose-response curve and the corresponding IC_50_ and IC_90_ values were generated by Graph-Pad Prism software (Graph-Pad soft ware, Inc.).

## SUPPLEMENTARY FIGURE AND TABLE


