# Supplementary material for: The AXL receptor tyrosine kinase is associated with adverse prognosis and distant metastasis in esophageal squamous cell carcinoma
Source: Oncotarget. 2016 May 9;7(24):36956–70. doi: 10.18632/oncotarget.9231 (PMC5095051; doi:10.18632/oncotarget.9231)
Supplement: Supplementary file 1 [file oncotarget-07-36956-s001.pdf]

The AXL receptor tyrosine kinase is associated with adverse prognosis and distant metastasis in esophageal squamous cell carcinoma

SUPPLEMENTARY FIGURE AND TABLE

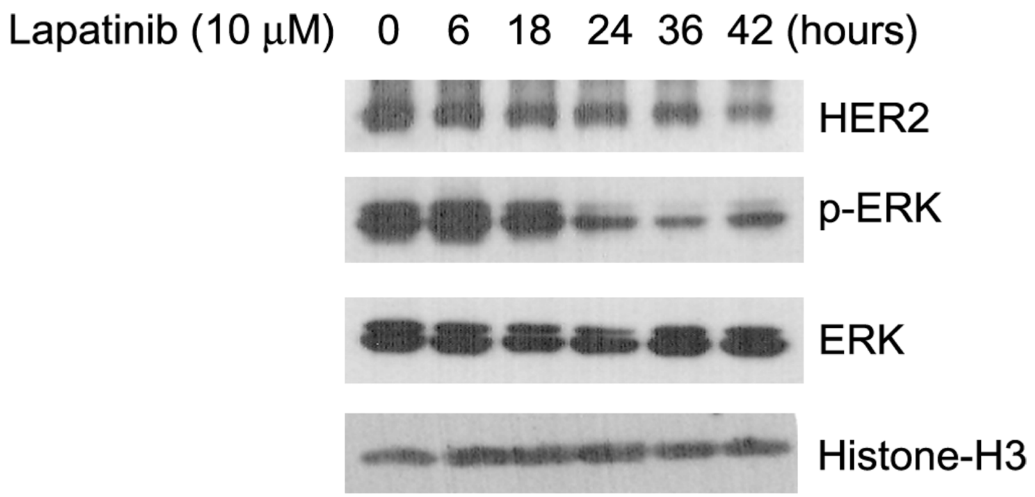

Supplementary Figure S1: HER2, phosphor-ERK (p-ERK), and ERK expression in ESCC cells treated with lapatinib (10 µM) for the indicated time periods. Histone H3 served as a loading control.

Supplementary Table S1: Association of CCRT treatment and expression of AXL and HER2 in ESCC

	CCRT		*p*-value
	**No**	**Yes**	
**Tumor_AXL expression**			0.446
Negative	6 (15.8)	17 (21.8)	
Positive	32 (84.2)	61 (78.2)	
**Tumor_HER2 expression**			1.000
Negative	37 (82.2)	72 (81.8)	
Positive	8 (17.8)	16 (18.2)	
